# Real-World Experience With Tofacitinib in Steroid-Dependent Patients With Moderate-to-Severe Ulcerative Colitis on Immunomodulators

**DOI:** 10.7759/cureus.79456

**Published:** 2025-02-22

**Authors:** Rajendra Bhati, Abhishek Yadav, Bobby Mitrolia, Vivek Saini, Sunil K Dadhich, Narendra Bhargava

**Affiliations:** 1 Gastroenterology and Hepatology, All India Institute of Medical Sciences, Jodhpur, Jodhpur, IND; 2 Gastroenterology and Hepatology, Dr. Sampurnanand Medical College, Jodhpur, IND

**Keywords:** biologics agents, immunomodulators, inflammatory bowel disease (ibd), tofacitinib, ulcerative colitis (uc)

## Abstract

Introduction: Patients with moderate-to-severe ulcerative colitis (UC) who are steroid-dependent despite being on immunomodulators pose significant clinical challenges. Currently, biologics therapy is the only option apart from considering surgery. Tofacitinib is an oral Janus kinase inhibitor used as a second-line therapy for patients with UC following the failure of biologics therapy. There is a lack of Indian data on the use of tofacitinib in biologically naïve patients with moderate-to-severe UC. Our study aimed to evaluate the efficacy and safety of tofacitinib in steroid-dependent patients with moderate-to-severe UC who were already on immunomodulators.

Materials and methods: An open-label, single-arm, prospective observational study was conducted on steroid-dependent UC patients from January 2021 to June 2023. All eligible patients received tofacitinib according to standard guidelines. Clinical response and remission were assessed at eight and 24 weeks.

Results: A total of 53 patients met the inclusion criteria, of whom 52.83% were male and 47.16% were female. The mean age was 40.35 years (SD ± 10.85 years). At eight weeks, 36 patients (67.92%) showed a clinical response, 19 (35.8%) achieved clinical remission, and 12 (22.64%) attained endoscopic remission. At 24 weeks, 35 patients (66.03%) achieved clinical remission (p<0.001), and 22 (41.50%) attained endoscopic remission (p=0.003). A total of 36 patients (68%) showed a clinical response, while 17 (32%) did not respond to tofacitinib at eight weeks. CRP and Mayo scores showed significant differences between responders and non-responders (p<0.001). Only two patients developed herpes zoster infection, both of whom were managed conservatively without requiring discontinuation of tofacitinib. No other adverse effects, such as thromboembolic events, were observed during the study period.

Conclusion: The oral small-molecule tofacitinib is an effective and safe treatment for moderate-to-severe UC that is steroid-dependent in patients already on immunomodulators.

## Introduction

Ulcerative colitis (UC) is a chronic inflammatory disease of the large intestine that occurs in genetically susceptible individuals [1]. UC follows a protracted course of exacerbations and remissions and often requires lifelong therapy [2]. Steroids and biologics are the primary options for induction therapy in moderate to severe UC, while immunomodulatory drugs and biologics are necessary for maintaining remission in these patients [3].

Up to 15-20% of patients do not respond to steroids, and 20% may become steroid-dependent during the course of the illness [4]. This subgroup of UC patients requires steroid-sparing agents such as azathioprine, infliximab, adalimumab, and tofacitinib to maintain remission [4]. The response to biologics varies, with approximately 40% of patients not responding to induction therapy. Among those who do respond, nearly one-third lose their response during the follow-up period [5]. Additionally, in resource-limited countries like India, the affordability and availability of biologics remain significant concerns.

Although immunomodulatory drugs like azathioprine are frequently used as steroid-sparing agents for maintenance therapy, they have several side effects, including leukopenia, thrombocytopenia, hepatotoxicity, and a potential risk of malignancy [6]. Tofacitinib, a small molecule effective in rheumatoid arthritis, has demonstrated efficacy in moderate to severe UC patients for induction and maintenance therapy [7]. Tofacitinib is an orally administered drug that inhibits all Janus kinases (JAKs) but preferentially targets JAK1 and JAK3 [8].

Tofacitinib is often used as a substitute for biologics in cases of nonresponse, and most studies on tofacitinib predominantly include biologically experienced populations. However, data on biologically naïve UC patients treated with this drug are limited. The side effect profile of tofacitinib includes thromboembolic events, reactivation of varicella zoster, and hypertriglyceridemia, with additional concerns regarding its long-term safety in tuberculosis-endemic countries like India.

Tofacitinib is an oral, relatively affordable, and seemingly effective option for UC. However, data on the Indian population are scarce, and most studies are either underpowered or retrospective in nature. Therefore, we conducted a prospective study to evaluate the efficacy and safety of tofacitinib in moderate to severe, predominantly biologically naïve UC patients for induction and maintenance in a subgroup of steroid-dependent patients receiving immunomodulators.

## Materials and methods

Study design

An open-label, single-arm, prospective observational study was conducted on steroid-dependent UC patients enrolled in the Department of Gastroenterology from January 2021 to June 2023. The Institutional Ethics Committee of Dr. Sampurnanand Medical College approved the study (approval number: SNMC/IEC/IIP/2022/063).

Study population

The sample size was 53. The sample size for tofacitinib was calculated based on its expected efficacy in achieving remission rates in patients with UC, as reported in the OCTAVE trial [7]. The eligibility criteria were as follows: adult patients (≥18 years) with steroid-dependent moderate to severe UC (endoscopically and histologically confirmed) who were already on immunomodulators. Patients with a positive IgG varicella-zoster virus antibody and/or a past history of chickenpox, thromboembolic disorders, ischemic heart disease, latent or active tuberculosis, pregnancy, or immunocompromised status were excluded. Steroid dependence was defined as an inability to wean systemic steroids below 10 mg of prednisolone within three months without recurrent active disease or symptomatic relapse of inflammatory bowel disease within three months of stopping steroids [9].

Assessment of outcomes

The Mayo score includes four parameters: stool frequency, rectal bleeding, mucosal appearance on endoscopy, and physician rating of disease activity [10]. Each parameter is scored from 0 to 3, where 0 denotes normal, and 3 denotes the maximum disease activity grade. A higher total score indicates more severe disease activity. The sum of all four parameter scores was calculated, followed by an analysis of the parameters. Clinical response was defined as a decrease in the Mayo score by 3 points from baseline, with a sub-score of 0 for rectal bleeding. Clinical remission was defined as a Mayo score of <2, with no sub-score >1.

Treatment protocol

Before the initiation of tofacitinib, screening was conducted for latent tuberculosis using an interferon-gamma release assay, the Mantoux test, and contrast-enhanced CT of the chest. Additional tests included a complete blood count, lipid profile, human immunodeficiency virus, hepatitis B surface antigen, hepatitis B surface antibody, hepatitis C virus antibody, and varicella serology. Patients were induced with 40 milligrams of prednisolone once daily and 10 milligrams of tofacitinib twice daily. Prednisolone was tapered over four weeks and then withdrawn. Tofacitinib was administered at 10 milligrams twice daily for eight weeks, followed by 5 milligrams twice daily as a maintenance dose. The immunomodulatory drug, most commonly azathioprine, was discontinued at this point.

Data collection

Patients were initially called for follow-up every two weeks during the first two months and then every four weeks thereafter. At each visit, a complete blood count, CRP level, and physical global assessment were performed, and any serious adverse events, if present, were recorded. Colonoscopy was conducted at baseline, eight weeks, and 24 weeks. The Mayo score was calculated at baseline, eight weeks, and 24 weeks.

Study outcomes

The primary outcomes were clinical response, clinical remission, and endoscopic remission at eight and 24 weeks. The secondary outcome was the occurrence of serious adverse events, such as cardiovascular events, deep vein thrombosis, herpes infection, or reactivation of hepatitis B.

Statistical analysis

Data were analyzed using OpenEpi software (Dean AG, Sullivan KM, Soe MM. OpenEpi: Open Source Epidemiologic Statistics for Public Health, www.OpenEpi.com). Continuous data were reported as the normal distribution mean with standard deviation (SD). Categorical data were presented as frequencies and percentages. Pre- and post-treatment Mayo scores and CRP levels at baseline, eight weeks, and 24 weeks were compared using a paired t-test and ANOVA. A p-value of <0.05 was considered statistically significant.

## Results

Patient characteristics

A total of 553 patients presented with UC in our department during the study period, of whom 64 were steroid-dependent while on immunomodulators and had moderate to severe UC. However, only 53 patients met the inclusion criteria and provided informed consent. The mean age was 40.35 years (SD ± 10.85 years). Of these 53 patients, 28 (52.83%) were male, and 25 (47.16%) were female. A total of 39 patients (73.58%) had moderate disease activity, while 14 (26.41%) had severe activity. Nine patients had proctitis, 30 had left-sided colitis, and 14 had extensive colitis. The mean Mayo score and CRP level at baseline were 8.62 (SD ± 1.25) and 19.99 (SD ± 4.69), respectively. A total of 51 patients (96.22%) were on azathioprine and mesalamine, while two patients (3.77%) were on a combination of adalimumab and azathioprine. Baseline characteristics are shown in Table 1.

**Table 1:** Baseline characteristics E1, E2, and E3 denote the extent of the disease according to the Montreal classification. CRP: C-reactive protein, SD: standard deviation, E1: proctitis, E2: left side colitis, E3: extensive colitis

Total number of patients	53
Mean age	40.35 years (SD ± 10.85)
Sex	
Male	28 (52.83%)
Female	25 (47.16%)
Mean CRP score	19.99 mg/L (SD ± 4.69)
Mean Mayo score	8.62 (SD ± 1.25)
Mayo endoscopic sub-score	
1 (mild)	0
2 (moderate)	39 (73.58%)
3 (severe)	14 (26.41%)
Previous treatment	
Azathioprine + mesalamine	51 (96.22%)
Azathioprine + adalimumab	2 (3.77%)
Duration of disease	
≤2 years	9 (16.98%)
2-5 years	25 (47.16%)
5-10 years	16 (30.18%)
≥10 years	3 (5.66%)
Extent of disease	
E1	9 (16.98%)
E2	30 (56.60%)
E3	14 (26.41%)

Primary outcomes

At eight weeks, 36 (67.92%) patients showed a clinical response, 19 (35.8%) patients achieved clinical remission, and 12 (22.64%) patients attained endoscopic remission. Patients who exhibited a clinical response to tofacitinib at eight weeks were considered for further study. One patient showed improvement at eight weeks but failed to respond at 20 weeks; therefore, at 24 weeks, they were considered non-responsive. At 24 weeks, 35 (66.03%) patients achieved clinical remission (p<0.001), and 22 (41.50%) patients attained endoscopic remission (p=0.003) (Table 2).

**Table 2:** Primary outcomes at eight and 24 weeks * statistically significant at p<0.05

Primary outcomes	8 weeks		24 weeks		p-value (McNemar's)
	No.	%	No.	%	
Clinical response	36	67.92	35	66.03	0.475
Clinical remission	19	35.8	35	66.03	<0.001*
Endoscopic remission	12	22.64	22	41.50	0.003*

Secondary outcomes

Only two patients developed herpes zoster infection in the second week of tofacitinib induction. These patients were managed conservatively and did not require discontinuation of tofacitinib. No other adverse effects, such as thromboembolic events, were observed during the study period. The mean CRP at baseline was 19.36 ± 4.62, significantly decreasing to 2.92 ± 1.31 at eight weeks and 1.46 ± 1.20 at 24 weeks. Similarly, the mean Mayo score at baseline was 8.61 ± 1.31, which significantly decreased at eight weeks (2.92 ± 1.31) and at 24 weeks (0.91 ± 1.10) (Table 3).

**Table 3:** CRP and Mayo scores in the responder group CRP: C-reactive protein, ANOVA: analysis of variance

			p-value (repeated measures ANOVA test)
CRP score (mg/L)	Baseline	19.36 ± 4.62	<0.001
	8 weeks	2.92 ± 1.31	
	24 weeks	1.46 ± 1.20	
Mayo score	Baseline	8.61 ± 1.31	<0.001
	8 weeks	2.58 ± 1.42	
	24 weeks	0.91 ± 1.10	

Non-responder group

The mean CRP at baseline was 21.33 ± 4.70, significantly reducing to 5.26 ± 1.02 at eight weeks. Similarly, the mean Mayo score at baseline was 8.64 ± 1.16, significantly decreasing to 6.82 ± 0.92 at eight weeks. Despite the significant reduction in biochemical and clinical parameters, CRP failed to drop below 5, and the Mayo score reduction was less than 2 points, thereby not meeting the clinical response criteria.

Comparison of tofacitinib responder and non-responder groups

Thirty-six (68%) patients clinically responded, while 17 (32%) did not respond to tofacitinib at eight weeks. The two groups showed no significant difference in baseline CRP and Mayo scores. However, after eight weeks of tofacitinib treatment, CRP and MAyo scores showed significant differences between the responder and non-responder groups (p<0.001) (Table 4).

**Table 4:** Comparison of tofacitinib responder and non-responder groups * statistically significant at p<0.05 CRP: C-reactive protein, SD: standard deviation

		Responders (mean ± SD)	Non-responders (mean ± SD)	p-value (t-test)
Total number of patients (53)		36 (68%)	17 (32%)	
CRP score (mg/L)	Baseline	19.36 ± 4.62	21.33 ± 4.70	0.162
	8 weeks	2.92 ± 1.31	5.26 ± 1.02	<0.001*
Mayo score	Baseline	8.61 ± 1.31	8.64 ± 1.16	0.921
	8 weeks	2.58 ± 1.42	6.88 ± 0.92	<0.001*

## Discussion

This study assessed the effectiveness and safety of tofacitinib in the induction and maintenance of steroid-dependent UC patients with moderate-to-severe activity while on immunomodulators. The OCTAVE trial conducted a detailed analysis of the induction and maintenance of tofacitinib in UC [11]. The standard dose of tofacitinib is 10 mg twice daily for induction, followed by 5 mg twice daily for maintenance [11]. Similarly, in our study, tofacitinib was administered at 10 mg twice daily for induction, followed by 5 mg twice daily as a maintenance dose, with a minimum duration of 24 weeks.

Our study's mean age was 40.35 years, and 52.83% of the participants were male. Patients with moderate UC constituted 73.58% of the total study population, while 26.41% had severe disease activity. The mean Mayo score at baseline was 8.62, and 96.22% of the patients were biologically naïve.

After eight weeks of therapy, 67.92% of patients exhibited a clinical response, 35.8% achieved clinical remission, and endoscopic remission was observed in 22.64% of patients. The primary non-response rate at eight weeks was 32% (17/53). Tofacitinib was continued at 5 mg twice daily for 24 weeks for those who achieved a clinical response at eight weeks. By the end of 24 weeks, 66.03% of the total enrolled patients (n=53) achieved clinical remission, and 41.50% attained endoscopic remission. This suggests that more patients eventually achieved clinical and endoscopic remission when the drug was continued for 24 weeks. However, one patient (1/35) experienced a loss of response during follow-up and subsequently required rescue therapy. In total, 18 patients (17+1) experienced treatment failure by week 24.

Our study's clinical remission and response rates are comparable to those reported by Singh et al., who achieved clinical remission and response rates of 41.8% and 65.1% at eight weeks with tofacitinib in moderately active UC [11]. However, their study population included only patients with moderate UC, whereas our population also included 29% of patients with severe disease. Furthermore, follow-up data for 24 weeks were unavailable in the above study. A recent study by Giri et al. reported a clinical remission rate of 59.6% (28/47) at 24 weeks with tofacitinib in patients with moderate to severe active UC. However, in that study, a higher dose of tofacitinib (10 mg twice daily for 16 weeks) was used to achieve clinical remission. In contrast, our study demonstrated that patients achieved clinical remission with the conventional dosing regimen of 5 mg twice daily for a longer duration [12].

Similar findings were also reported by Taxonera et al. [5] in a meta-analysis of 17 real-world tofacitinib studies comprising 1,162 moderate UC patients. The study showed that clinical response at eight weeks, 12-16 weeks, and 24 weeks was achieved in 62.1%, 64.2%, and 50.8% of cases, respectively, while clinical remission was achieved in 34.7% of patients at eight weeks, 47% at 12-16 weeks, and 38.3% at 24 weeks. These findings suggest that more patients can achieve clinical remission if tofacitinib is administered for an extended period.

In our study, roughly 23% of patients achieved endoscopic remission at eight weeks, which increased to 41% at 24 weeks. In the aforementioned meta-analysis by Taxonera et al., mucosal healing was achieved in 48.3% and 45.3% of patients at eight weeks and 12-16 weeks, respectively [5]. The slightly lower rates observed in our study can be explained by the presence of 28% of patients with severe UC. Our study demonstrated similar remission and response rates at eight weeks of therapy; however, clinical and endoscopic remission rates continued to increase with the duration of treatment.

In another study by Ishida et al., the Mayo endoscopic score significantly improved post-treatment (p=0.03), and clinical response was observed in 77.3% and 72.4% of patients at one and six months, respectively [13]. The reduction in the mean Mayo score in our study from baseline (8.61 ± 1.31) to 2.58 ± 1.42 at eight weeks is comparable to the OCTAVE study, in which the Mayo score decreased from 9.0 ± 1.4 to 3.4 ± 1.8.7. The change in the mean Mayo score in the responder and non-responder groups was -6.02 and -1.76, respectively, at eight weeks of induction therapy. While the change in the Mayo score was significant in both groups, it was <2 in the non-responder group.

Although CRP is the most commonly used biomarker in the clinical management of inflammatory bowel disease, it is a non-specific marker that reflects systemic inflammation. A post hoc analysis showed early decreases in CRP were associated with achieving efficacy outcomes following tofacitinib 10 mg twice daily induction therapy in the UC clinical program [14]. Our study also found a clinically significant (p<0.005) decrease in CRP from baseline to eight weeks of induction therapy in the responder group compared to the non-responder group.

In the OCTAVE trial, an increased incidence of herpes zoster infection was observed with a higher dose of tofacitinib (10 mg) compared to a lower dose (5 mg) (5.1% vs. 1.5%). In contrast, in the placebo group, the incidence was 0.5%. Cardiovascular events occurred in five patients in the tofacitinib group, while none were reported in the placebo group [7]. A systematic review conducted by Taxonera et al. showed no cardiovascular or major thromboembolic complications, while herpes zoster was reported in 3.4% of cases [5]. However, in our study, 3.77% of patients had herpes zoster, and no serious adverse events occurred.

Concerning tuberculosis reactivation concerns, our study observed no tubercular reactivation cases. However, a recently published study by Giri et al. reported tuberculosis reactivation in two out of 47 patients despite negative screening results [12]. Tuberculosis is the most common opportunistic infection (26/5671) in tofacitinib-treated rheumatoid arthritis patients, with a median time between the start of the drug and diagnosis of 64 weeks (range: 15-161 weeks) [15]. Therefore, a long-term follow-up, along with a larger study population of tofacitinib use in UC, is likely needed to better predict the risk of tuberculosis reactivation in endemic areas.

Our study is one of the first to demonstrate the efficacy and safety of tofacitinib in steroid-dependent, moderate-to-severe UC patients receiving immunomodulators. It was prospective in nature, with a long follow-up, and endoscopic remission was evaluated in subsequent follow-ups. In our study, most patients were biologically naïve, suggesting that tofacitinib can be used as a first-line advanced therapy for steroid-dependent, moderate-to-severe UC patients in resource-limited countries.

However, this study had some limitations. First, the sample size was small. Second, it was a single-arm study with no placebo group. Third, it was conducted at a single center.

## Conclusions

Oral small-molecule tofacitinib is an effective and safe alternative for steroid-dependent, moderate-to-severe UC patients already on immunomodulators. Tofacitinib can precede biologics as a first-line advanced therapy in this patient population. Prolonged therapy helps achieve remission in patients who show a clinical response at eight weeks.

## References

[1] Abraham C, Cho JH (2009). Inflammatory bowel disease. N Engl J Med.

[2] Kayal M, Shah S (2019). Ulcerative colitis: current and emerging treatment strategies. J Clin Med.

[3] Raine T, Bonovas S, Burisch J (2022). ECCO guidelines on therapeutics in ulcerative colitis: medical treatment. J Crohns Colitis.

[4] Faubion WA Jr, Loftus EV Jr, Harmsen WS, Zinsmeister AR, Sandborn WJ (2001). The natural history of corticosteroid therapy for inflammatory bowel disease: a population-based study. Gastroenterology.

[5] Taxonera C, Olivares D, Alba C (2022). Real-world effectiveness and safety of tofacitinib in patients with ulcerative colitis: systematic review with meta-analysis. Inflamm Bowel Dis.

[6] Yewale RV, Ramakrishna BS, Doraisamy BV (2023). Long-term safety and effectiveness of azathioprine in the management of inflammatory bowel disease: a real-world experience. JGH Open.

[7] Sandborn WJ, Su C, Sands BE (2017). Tofacitinib as induction and maintenance therapy for ulcerative colitis. N Engl J Med.

[8] Danese S, Grisham M, Hodge J, Telliez JB (2016). JAK inhibition using tofacitinib for inflammatory bowel disease treatment: a hub for multiple inflammatory cytokines. Am J Physiol Gastrointest Liver Physiol.

[9] Dignass A, Eliakim R, Magro F (2012). Second European evidence-based consensus on the diagnosis and management of ulcerative colitis part 1: definitions and diagnosis. J Crohns Colitis.

[10] Schroeder KW, Tremaine WJ, Ilstrup DM (1987). Coated oral 5-aminosalicylic acid therapy for mildly to moderately active ulcerative colitis. A randomized study. N Engl J Med.

[11] Singh A, Midha V, Kaur K (2024). Tofacitinib versus oral prednisolone for induction of remission in moderately active ulcerative colitis [orchid]: a prospective, open-label, randomized, pilot study. J Crohns Colitis.

[12] Giri S, Bhrugumalla S, Kamuni A (2024). Upfront tofacitinib in patients with biological-naïve ulcerative colitis - an Indian multicentric experience. Indian J Gastroenterol.

[13] Ishida N, Miyazu T, Tamura S (2022). Real-world efficacy and safety monitoring for predicting continuation of tofacitinib therapy in patients with ulcerative colitis. Dig Dis Sci.

[14] Dubinsky MC, Magro F, Steinwurz F (2023). Association of C-reactive protein and partial Mayo score with response to tofacitinib induction therapy: results from the ulcerative colitis clinical program. Inflamm Bowel Dis.

[15] Winthrop KL, Park SH, Gul A (2016). Tuberculosis and other opportunistic infections in tofacitinib-treated patients with rheumatoid arthritis. Ann Rheum Dis.

